# Supplementary material for: Long-Term Impact of Deep Brain Stimulation in Parkinson’s Disease: Does It Affect Rehabilitation Outcomes?
Source: Medicina (Kaunas). 2024 Jun 1;60(6):927. doi: 10.3390/medicina60060927 (PMC11205741; doi:10.3390/medicina60060927)
Supplement: Supplementary file 1 [file medicina-60-00927-s001.zip › medicina-2987898-supplementary.pdf]

Supplementary Table S1. Correlation analysis between Age and and 6-minute walk test (6MWT), Timed Up and Go (TUG), Time from Intervention, and Time between Diagnosis and DBS

Control Variables			***6MWT (T1-T0)***	***TUG (T1-T0)***	***Time from Intervention***	***Time between Diagnosis and DBS***	***Age***
None	6MWT (T1-T0)	*Correlation (r)*	1.000	-0.421	-0.516	-0.246	-0.427
		p Value	<0.0001	0.051	0.014	0.270	0.047
	TUG (T1-T0)	*Correlation (r)*	-0.421	1.000	0.021	0.434	0.031
		p Value	0.051	<0.0001	0.926	0.044	0.890
	Time from Intervention	*Correlation (r)*	-0.516	0.021	1.000	-0.075	0.504
		p Value	0.014	0.926	<0.0001	0.739	0.017
	Time between Diagnosis and DBS	*Correlation (r)*	-0.246	0.434	-0.075	1.000	-0.058
		p Value	0.270	0.044	0.739	<0.0001	0.799
	Age	*Correlation (r)*	-0.427	0.031	0.504	-0.058	1.000
		p Value	0.047	0.890	0.017	0.799	<0.0001
Age	6MWT (T1-T0)	*Correlation (r)*	1.000	-0.452	-0.384	-0.299	
		p Value	<0.0001	0.040	0.085	0.187	
	TUG (T1-T0)	*Correlation (r)*	-0.452	1.000	0.006	0.436	
		p Value	0.040	<0.0001	0.979	0.048	
	Time from Intervention	*Correlation (r)*	-0.384	0.006	1.000	-0.054	
		p Value	0.085	0.979	<0.0001	0.817	
	Time between Diagnosis and DBS	*Correlation (r)*	-0.299	0.436	-0.054	1.000	
		p Value	0.187	0.048	0.817	<0.0001	

Abbreviations: DBS: Deep Brain Stimulation; TUG: Time Up and Go; 6MWT: 6-Minutes Walking Test; T0: Baseline; T1: After rehabilitation.
